# Rates, patterns, and predictors of complementary medicine use among patients with musculoskeletal diseases

**DOI:** 10.1371/journal.pone.0287337

**Published:** 2023-06-23

**Authors:** Fatima Alnaimat, Hamza Alduraidi, Laila Alhafez, Lujain Abu Raddad, Bassem I. Haddad, Mohammad Hamdan, Jihad Alajlouni, Fatma U. Afifi

**Affiliations:** 1 Division of Rheumatology, Department of Internal Medicine, School of Medicine, University of Jordan, Amman, Jordan; 2 School of Nursing, The University of Jordan, Amman, Jordan; 3 Aqaba Medical Sciences University, Aqaba, Jordan; 4 School of Medicine, The University of Jordan, Amman, Jordan; 5 Division of Orthopedics, Department of Special Surgery, School of Medicine, University of Jordan, Amman, Jordan; 6 Department of Pharmaceutical Chemistry and Pharmacognosy, Applied Science Private University, Amman, Jordan; King Saud University, SAUDI ARABIA

## Abstract

**Aim:**

To investigate the extent of complementary medicine (CM) use and the most common therapies utilized by Jordanian patients with musculoskeletal (MSK) diseases.

**Methods:**

A semi-structured questionnaire was used to conduct a cross-sectional survey of outpatient orthopedic and rheumatology patients at an academic medical center in Amman, Jordan between January and September 2020.

**Results:**

A convenience sample of 1001 patients was interviewed (82% females). Pearson’s chi-square comparisons showed that nutritional CM was used by 43.4% of patients, while 29.8% used physical CM, and 16% used both. Almost all used the nutritional or physical CM in addition to their prescribed treatment. Nutritional form use was significantly higher among females, older age groups, married people, and those who worked (p < .05). Physical form use was statistically more prevalent in older age groups and those with a higher level of education (p < .05). Family income and urban residence were not significantly associated with the use of either form of CM therapy. Olive oil was the most frequently reported nutritional type (22.9%), and cupping was the most reported physical type (41.6%). Recommendations to use CM came primarily from family members or friends (64% of nutritional CM users and 59% of physical CM users). A physician or pharmacist was cited more frequently with physical CM (24% versus 8% for the nutritional form). In contrast, media sources were cited more for nutritional than physical form (28% versus 7%). Over half of the patients believed they received the desired effect from CM. Surprisingly, only 9.5% of the patients admitted to discussing their CM use with their physician.

**Conclusion:**

CM use is prevalent among Jordanian patients with MSK disorders. Most patients rely on family and friends for recommendations, and they rarely inform their physician of the CM use. Physicians should routinely inquire about CM to provide patients with information regarding their benefits and risks.

## Introduction

The World Health Organization defines complementary medicine (CM) as a large group of healthcare practices not indigenous to a country and not integrated into its dominant healthcare system [1]. Alternative medicine is frequently substituted for conventional medicine, whereas complementary medicine is typically used in conjunction with conventional medicine [2]. According to the 2019 WHO global report on traditional and complementary medicine, at least 80% of WHO Member States use traditional and CM, primarily in the Eastern Mediterranean, South-East Asia, and Western Pacific regions [3].

The National Center for Complementary and Integrative Health (NCCIH) divides complementary therapies into three delivery-based categories: nutritional (including dietary supplements, herbs, and special diets); psychological (including meditation and prayer); and physical (such as massage therapy), or combinations of any or all of the three types [2]. Traditional healers, Ayurvedic medicine, traditional Chinese medicine, homeopathy, naturopathy, and functional medicine may not fit into any of the above-mentioned categories.

Over 150 diseases and conditions can cause impairments in the muscles, bones, joints, and adjacent connective tissues [4]. Musculoskeletal disorders (MSK) are linked to decreased productivity, significant disability, and a lower quality of life [5].

Chronic musculoskeletal pain management is still a significant healthcare challenge. Both opioid and non-opioid analgesia have drawbacks and adverse effects in clinical practice [6]. CM use in MSK conditions has been addressed in many studies, and research in this area is growing steadily [6–10]. Arthritis is one of the most common conditions for which CM is used [11]. Healthcare professionals who utilized CM modalities themselves were more inclined to recommend them to their patients [12]. In a study of 320 Iranian healthcare professionals, 86.6% of respondents reported using at least one CM treatment [13]. Therefore, healthcare providers must possess complete and accurate information regarding their patients’ CM behaviors to properly counsel their patients on the potential benefits and risks of CM.

This study examines the prevalence, trends, and determinants of CM usage among patients with MSK illnesses. The questionnaire also seeks to determine the source of CM advice and whether patients have addressed their usage of CM with their physicians. No prior research has examined the prevalence of CM use among patients with these conditions in Jordan.

## Materials and methods

A cross-sectional survey was performed of outpatients attending the orthopedic and rheumatology clinic at the Jordan University Hospital (JUH) in Amman, Jordan. The hospital is a teaching hospital and is a tertiary referral center that serves large areas of the country, with over 400,000 outpatient visits in 2020 [14].

Patients were approached in the waiting rooms of the outpatient clinics by the research assistant who is a trained pharmacist, while waiting to be called for their doctor’s appointment. Interviews were conducted on weekdays during the morning clinics. The recruitment period ran from January to September of 2020, with an interruption between late March and mid-May of 2020 owing to the COVID-19 pandemic lockdown. The sampling frame of the study included all patients of JUH’s rheumatology and orthopedic outpatient clinics during the time period between January and September 2020. Sample frame members were approached, and those who agreed to participate were recruited using convenience sample technique, until the desired sample size was accomplished near the end of September 2020.

Sample size was calculated using Cochran’sSampling Techniques for cross-sectional surveys with proportional outcomes [15], where type 1 error margin, alpha, was set at .05, expected proportion of the population, p, was set at .50, and absolute error or precision, d, was set at .05. The calculation yielded a minimum required sample size of 901 subjects. An additional 10% were recruited to count for potential missing data, resulting in recruiting a total sample size of 1,001 subjects. Inclusion criteria were any outpatient client aged 16 or older who can write and read simple Arabic and is willing to participate in the study. Child clients aged 11–16 years were accompanied by a next-of-kin adult. No specific criteria were applied to exclude participants besides the inability to give informed consent. Males and females of various ages were approached to ensure a representative cross-sectional sample of musculoskeletal disorders. Patients were not compensated for their time, but they were told they could refuse to answer any questions that made them feel uneasy. None of the physicians involved in each patient’s care were present during the interview.

This study’s questionnaire, developed from past research on patients with cancer, diabetes, and chronic medical conditions conducted in Jordan by one of the co-authors, was confirmed to be valid for Jordanian patients in three prior studies [16–18]. Table 1 shows the main aspects of the questionnaire; the full questionnaire can be accessed in S1 File of this study. Each question had a predetermined response, and the interviews lasted approximately 20 minutes.

**Table 1:** Key aspects of the study’s questionnaire.

Theme	Details
**Demographic data**	Gender, age group, marital status, next of kin, occupation, area of residence, educational level, income
**Medical history**	Musculoskeletal diagnoses and other medical problems.
**Details of CM use**	A-Nutritional (Oral).
	B-Physical
**Source of recommendation to use the CM**	Family, friends, physicians, pharmacists, or the media.
**The outcome of CM use**	Satisfaction with CM therapy, communication with the treating physician regarding CM use

For this study, the CM approaches were divided into nutritional and physical. A pilot survey was conducted on 100 patients to assess the questionnaire’s applicability in the MSK sample. No changes were made following the pilot study.

The study, following the guidelines laid out by the Declaration of Helsinki, was approved by the local Institutional Review Board at Jordan University Hospital (JUH) and the Scientific Committee at The University of Jordan’s Deanship of Scientific Research.

### Statistical analysis

The data was entered and analyzed using IBM’s SPSS software, version 26. Descriptive statistics, such as counts, percentages, and graphs, were used to present sample characteristics and rates of CM use both orally and physically, as well as the description of materials used in each type of CM. Associations between CM type and categorical sample characteristics, such as gender, marital status, employment status, and educational level, were tested using the non-parametric inferential statistical test of Pearson’s chi-square. Finally, in inferential statistics, alpha was determined at .05 to define statistical significance with a .95 level of confidence.

## Results

### Sample characteristics

The study included 1,001 patients with MSK diseases who visited clinics at JUH; 821 (82%) were female and 180 (18%) were male. Table 2 outlines the details of the patients’ demographic data. Although patients were of various age groups, the majority were above 40 years old. Most members of the sample (64.9%) were married. As for educational level, 41.8% of the sample had bachelor’s degrees or higher, while only 3.5% were illiterate. Most of the patients (79.6%) were unemployed at the time of data collection, and most were residing in urban areas (78.8%). Regarding monthly family income, the majority (56.4%) were in the middle-income range of JD 400–1,000 (1 JD = 1.40 USD) per month (Table 2). Osteoarthritis (22.1%) was the most prevalent musculoskeletal disease, followed by inflammatory arthritis (16.5%), and chronic myofascial pain/fibromyalgia (12.8%).

**Table 2:** Sample characteristics (N = 1,001).

Variable		Frequency (%)
Gender	Male	180 (18)
	Female	821 (82)
Age	<16	46 (4.6)
	16–25	63 (6.3)
	26–40	144 (14.4)
	41–50	161 (16.1)
	51–60	282 (28.2)
	>60	303 (30.3)
Marital Status	Single	212 (21.2)
	Married	649 (64.9)
	Divorced	18 (1.8)
	Widow	121 (12.1)
Employment	Working	204 (20.4)
	not working	796 (79.6)
Area of residence	Urban	788 (78.8)
	Rural	212 (21.2)
	Illiterate	35 (3.5)
	Elementary school	321 (32.1)
	High school	227 (22.7)
	College Degree or Higher	418 (41.8)
Monthly Income	<400 JD	389 (38.9)
	400–1000 JD	564 (56.4)
	>1000 JD	47 (4.7)
Diagnosis	Osteoarthritis	221 (22.1)
	Inflammatory Arthritis	165 (16.5)
	Myofascial pain syndrome/fibromyalgia	128 (12.8)
	Injury/Fracture	95 (9.5)
	Connective Tissue Disease	57 (5.7)
	Osteoporosis	27 (2.7)
	Other	85 (8.5)
	Not Yet Diagnosed	203 (20.3)
Diagnosis Duration	< 1year	177 (17.7)
	1–5 years	312 (31.2)
	> 5 years	187 (18.7)
	First Visit	322 (32.2)

### Complementary medicine use among the study’s participants

Of the sample members, 433 (43.3%) used complementary nutritional medicine, while 298 (29.8%) used complementary physical medicine. While 159 (16%) people used both types of CM, 427 (43%) did not use any CM therapy.

The most common type of nutritional CM used by the patients was olive oil (52.4%), followed by Lepidium sativum (cress) (21.5%), collagen (11.5%) and Nigella sativa(7.9%). Details of the complementary medicine used by the study sample can be seen in Table 3. Most of those who reported using nutritional CM used only one type (61.3%), while 23.5% used two types, 9.7% used three types, and 0.9% used four or more types simultaneously.

**Table 3:** Complementary medicine (CM) therapies used by the study sample.

Frequency			Percentage	
**Physical CM (n = 298)**				
Cupping	124	41.6%	
Water therapy	72	24.2%	
Massage	38	12.8%	
Acupuncture	15	5%	
Bees stings	8	2.7%	
Dead sea mud	4	1.34%	
Nutritional CM (n = 433) *				
Olea europaea (Common Olive)-oil	227	52.4%	
Lepidium sativum (Cress)	93	21.5%	
Collagen	50	11.5%	
Nigella sativa	34	7.9%	
Curcuma longa(turmeric)	34	7.9%	
Fish oil	39	9%	
Linum usitatissimum (flax or linseed)	28	6.5%	
Zingiber officinale (ginger)	28	6.5%	
Honey	20	4.6%	
Glucosamine	17	3.9%	
Sesamum indicum(Sesame)- oil	12	2.8%	
Trigonella foenum-graecum (fenugreek)	8	1.8%	

*Percentages more than 100% due to some patients’ use of multiple types.

Cupping was the most prevalent physical modality used by the study participants(41.6%), followed by water therapy (24.2%), massage (12.8%) and acupuncture (5%) (Table 3). The vast majority (89.2%) of those who used complementary physical medicine only used one type, while 10.7% used two and 0.1% used three.

Most recommendations to use CM therapy came from family or friends (64% for nutritional CM and 59% for physical CM). In the case of physical CM, more patients cited a physician or pharmacist as the source of recommendations (8% for nutritional CM and 24% for physical CM). Almost all (95%) used nutritional or physical CM in addition to their prescribed treatment.

#### Socio-demographic characteristics of complementary medicine users

Nutritional CM use was significantly more prevalent among females, older age groups, married individuals, employed individuals, and orthopedic patients (p < .05), as seen in Table 4. furthermore, older age groups and those with a higher level of education reported physical CM use significantly more frequently (p < .05). Family income, residence area, and diagnosis duration were not significantly associated with either type.

**Table 4:** Socio-demographic characteristics of oral and physical CM users (N = 1,001).

Characteristic		Nutritional CM Use				Physical CM Use			
		Yes %	No %	Chi^2^ *(df)*	*p*	Yes %	No %	Chi^2^ *(df)*	*p*
Gender	male	27.2	72.8	23.263 *(1)*	.000*	28.3	71.7	.247 *(1)*	.344
	female	46.9	53.1			30.2	69.8		
Age group	<16	9	91	53.042 *(5)*	.000*	4.3	95.7	20.030 *(5)*	.003*
	16–25	30	70			20	80		
	26–40	31	69			25	75		
	41–50	44	56			29.2	70.8		
	51–60	54.6	45.4			32.3	67.7		
	>60	46.2	53.8			35	65		
Marital status	single	30	70	24.387 *(3)*	.000*	22.6	77.4	7.396 *(3)*	.060
	married	46.6	53.4			32	68		
	divorced	28	72			39	61		
	widow	52	48			30	70		
Residence area	urban	43	57	.630 *(1)*	.237	30.5	69.5	.810 *(1)*	.208
	rural	46	54			27.4	72.6		
Educational level	illiterate	45.7	54.3	6.287 *(3)*	.507	22.9	77.1	15.018 *(3)*	.036*
	elementary	40	60			23.7	76.3		
	high school	41	59			32	68		
	college or higher	44.7	45.3			34.1	65.9		
Employment	works	45	55	3.884 *(1)*	.029*	29.9	70.1	.001 *(1)*	.527
	does not work	37.3	62.7			29.9	70.1		
Family monthly income	< JD 400	41.4	58.6	1.424 *(2)*	.491	27.8	72.2	2.184 *(2)*	.336
	JD 400–1,000	45	55			31.7	68.3		
	> JD 1,000	40.4	59.6			25.5	74.5		
Clinic specialty	rheumatology	40	60	5.469 *(1)*	.011*	28	72	1.583 *(1)*	.117
	orthopedic	47	53			31.6	68.4		
Diagnosis duration	< 1 year	46.4	53.6	5.210 *(2)*	.157	24.6	75.4	6.698 *(2)*	.082
	1–5 years	49	51			32.9	67.1		
	> 5 years	39	61			36.3	63.7		

CM: Complementary Medicine

* Statistically significant; p <0.05

#### The outcome of complementary medicine use in the study sample

Half of the patients (53.8%) reported using CM therapies to alleviate their disease’s symptoms, 14% to improve their overall health, and 16% to slow the progression of their condition, while 10.2% of the patients believed that complementary therapy would cure their illness and 6% thought that it would assist in reducing the side effects of their regular medications. Fifty-five percent of the patients believed that they obtained the sought result from complementary therapy, compared to 45% who did not. Only 9.5% of patients reported discussing their CM use with their treating physician.

## Discussion

A growing number of patients are turning to CM [10]. The burden of MSK diseases has increased substantially from 2000 to 2015, with MSK diseases being the second leading cause of years lived with disability worldwide [19], Possible explanations might be the current increase in average life expectancy coupled with the fact that many degenerative MSK diseases are largely irreversible [20]. Our study found CM approaches to be a common practice among Jordanian patients with MSK disorders. Of the surveyed patients with MSK diseases, 43.4% showed a much higher prevalence of nutritional CM use compared to Jordanian patients with diabetes (16.6%) and hypertension (11.6%) [21].

The common use of CM among patients with MSK disorders worldwide has been previously reported [6–9, 11]. In a study of 200 Swedish patients, 29% indicated ongoing CM usage, while 65% had used CM at some point in their life [22]. Data from the 2012 National Health Interview Survey [23] showed that adult Americans with musculoskeletal pain used complementary health approaches significantly more than those without (41.6% vs 24.1%), with natural products used more frequently than mind-body approaches (24.7% vs 15.3%, respectively). Our study also demonstrated similar findings where nutritional CM was more common than physical CM or combined forms. This could be because oral medicine is self-administered, potentially less expensive, and more easily accessible [10, 24]. Almost all the study patients reported using nutritional or physical CM in conjunction with their prescribed treatment.

In this study, nutritional CM use was more common in females, married people, elders, and those who worked, which is consistent with the findings of other studies [6, 25, 26]. The increased likelihood of CM use in women may be related to their increased use of any medication (conventional or complementary) [27]. Females with other chronic diseases were shown to be the largest group of CM users in Jordan [21].

As religious, geographical, and cultural factors influence an individual’s propensity to seek CM [16], patients of different nationalities have different patterns of CM use for musculoskeletal disorders [26]. At 22.9%, olive oil was the most popular nutritional remedy. Extra-virgin olive oil is a popular food item in Mediterranean diets. It is rich in biologically active polyphenols, which have antioxidant and anti-inflammatory properties that may prevent osteoarthritis cartilage damage [28]. Some studies showed the potential role of olive extract in improving function and pain scores in osteoarthritis patients [29]. Adherence to a Mediterranean diet, which is rich in olive oil, was shown to have a potentially beneficial impact on the activity of axial spondyloarthropathy [30]. Lepidium sativum (cress) was the second most commonly reported nutritional CM. Cress is a fast-growing herb widely distributed in Jordan and many Asian countries [31]. Some research has shown that cress has anti-inflammatory, antioxidant, and immunomodulatory properties.

Cupping (hijama) was the most prevalent form of physical CM, a finding corroborated by other papers examining CM use in the region. In Jordan, 20.4% of patients with chronic diseases use cupping, a form of prophetic medicine prevalent in Arabic countries [21]; 61.4% of patients in Saudi Arabia were estimated to have used cupping [32]. Cupping has been used to treat a variety of conditions in many cultures. Although it is unknown exactly how cupping reduces pain, some suggest that the pain-gate theory, diffuse noxious inhibitory controls (DNICs), or reflex zone theory could provide possible explanations [33]. Multiple simultaneous complementary treatment approaches were not common in our patients as most patients use one type of complementary therapy.

Although family and friends were the major sources of recommendations for using nutritional and physical CM, physician recommendations were cited more frequently for physical CM compared to nutritional CM. Body-based practices are increasingly included in management guidelines for MSK disorders, such as osteoarthritis [34] and chronic back pain [35, 36]. For instance, treatments such as exercise, acupuncture, mindfulness-based stress reduction, cognitive behavioral therapy, and spinal manipulation are strongly recommended by the American College of Physicians to manage chronic low back pain [37].

Several studies [6, 26, 38] found a positive relationship between the use of CM and a higher education level. According to our findings, a higher level of education increases the likelihood of physical CM use but not nutritional CM. A previous study of Jordanian patients with chronic diseases also found no statistically significant correlation between education level and nutrional CM use [18].

Our study’s finding of increased CM use with the advancement of a participant’s age is consistent with previous research, as those older than 64 are more likely to use CM [6, 25]. This, however, might not always hold. When stratified by BMI, normal/underweight persons 50 years and older were less likely to use CM than those younger than 35 years [25].

The impact of financial factors on CM use has been variable. The World Bank classified Jordan as a lower-middle-income country in 2017 [39]. The poverty line in Jordan is defined by the 2008 Jordanian Household Expenditure and Income Survey as 400 JOD of monthly expenditure [40]. Reportedly 41.4% of participants whose family was below the poverty line used nutritional CM compared to 27.8% who used physical CM. Although this difference is not statistically significant, fewer patients from lower income categories turned to complementary physical modalities due to the higher cost inherent to those approaches. Our findings concord with Herman et al. that there is no association between using CM and income [41]. A study from India found a high proportion of CM use among those with low monthly income, which could be attributed to CM’s accessibility and affordability [42], as is the case in Jordan [17, 43] and other Arab countries [44] where certain plant-based preparations are deeply rooted in the tradition of disease treatment, making medicinal herbs more accessible and affordable. In contrast, more expensive supplements, such as glucosamine and chondroitin [41], are uncommon treatments in Jordan and were utilized by a negligible proportion of patients in this study.

In this study sample, most patients believed CM would relieve disease symptoms; in contrast, a minority thought the CM therapy would cure their disease. People with rheumatic disorders resort to CM for various reasons, including limited access to some treatments due to the chronicity of these diseases, high costs of standard therapies such as biologic medications, and rigorous regulations from insurance companies in addition to concerns about drug side effects [10]. Phang et al. analyzed 60 randomized controlled trials on using CM for rheumatic diseases [45]. They reported that certain CM therapies, such as acupuncture for osteoarthritis, may be advantageous for rheumatic disorders. However, those trials were diverse regarding CM interventions, disease indicators utilized to assess outcomes, and CM therapy efficacy. Similar observations were noted by Danve et al. on CM therapy research in patients with axial spondyloarthropathy [46].

Most study participants reported using CM in conjunction with conventional treatment. Given the high prevalence of CM use among Jordanian patients with musculoskeletal disorders observed in this study, there is always the possibility that some patients may conceal their preference for CM therapy and non-adherence to standard treatment, which could compromise the quality of their medical care. Therefore, the findings of this study serve as an important reminder for physicians treating patients with musculoskeletal disorders to be aware of their patients’ use of CM and to routinely inquire about it to provide appropriate guidance on its potential benefits and risks to their specific medical conditions. Kocyigit et al. reviewed a variety of complementary and alternative medicine (CM) treatments for ankylosing spondylitis, rheumatoid arthritis, and fibromyalgia [10]. The authors concluded that these therapies are safe procedures with some positive outcomes, but that there is a poor level of evidence for many CM strategies. This emphasizes the necessity for higher-quality research in this field to determine the efficacy of CM therapy.

Socio-demographic characteristics of CM users are closely related across different rheumatologic and orthopedic diagnoses [27, 47, 48]. In patients with osteoarthritis, higher education was specifically associated with increased use of glucosamine/chondroitin [27].

A striking observation from our study is the low communication rate of CM use with the treating physician. According to the results of a systematic review conducted to gain knowledge on the perspectives of rheumatologists on CM [49], the familiarity of the rheumatologist with CM therapy and the level of scientific evaluation of the therapy both play a role in shaping those perspectives.

## Limitations

The study’s strengths include recruiting a large number of Jordanian patients with musculoskeletal disorders for the first time and aiming to educate medical professionals on common health practices among patients with chronic conditions so that they can counsel patients on the risks versus benefits ratio. Like other cross-sectional studies, this research has limitations. Due to the single interview, lack of longitudinal follow-up, and reliance on patient self-reporting, it was impossible to determine the doses and duration of CM use objectively. Positive or negative CM effects on musculoskeletal disease severity were not assessed, thus limiting the influence of these practices on patients’ adherence to standard therapy, nor did the study permit the evaluation of the trends in the use of various complementary modalities in a specific musculoskeletal disease. In addition, because the current study was done on patients who attended specialized rheumatology and orthopedic clinics at an academic institution, the results cannot be applied to Jordanian patients with MSK issues who may not have access to specialized care.

## Conclusion

This study confirms a high prevalence of CM use among patients with musculoskeletal disorders in Jordan. The results indicate that women are more likely than men to use CM, and patients with a higher education level tend to use physical CM more frequently. Family and friends were the primary sources of nutritional and physical CM recommendations, whereas physicians recommended physical CM more often than nutritional CM. Almost all participants reported using CM in conjunction with their prescribed treatment. Still, it is important to note that some patients may conceal their non-adherence to standard therapy, which could jeopardize the quality of their medical care.

Consequently, these findings underscore the significance of physicians being aware of their patients’ CM practices and routinely inquiring about them to offer appropriate advice on CM’s potential benefits and drawbacks for their specific medical conditions. Given the high frequency of CM use demonstrated in this study, the question "Are you using any complementary medicine therapy?" should be a standard component of patients’ medical histories.

## Supporting information

S1 File (PDF)

## References

[1] WHO. WHO guidelines on developing consumer information on proper use of traditional, complementary and alternative medicine. Available: https://apps.who.int/iris/bitstream/handle/10665/42957/9241591706.pdf;jsessionid=AE99D5B660994A8EEA07FC042EB4F6BB?sequence=1

[2] Complementary, Alternative, or Integrative Health: What’s In a Name? | NCCIH. [cited 8 Nov 2022]. Available: https://www.nccih.nih.gov/health/complementary-alternative-or-integrative-health-whats-in-a-name

[3] Organization WH. WHO global report on traditional and complementary medicine 2019. Geneva PP—Geneva: World Health Organization; Available: https://apps.who.int/iris/handle/10665/312342

[4] Musculoskeletal health. [cited 8 Nov 2022]. Available: https://www.who.int/news-room/fact-sheets/detail/musculoskeletal-conditions

[5] Sangha O. Epidemiology of rheumatic diseases. Rheumatology. 2000;39: 3–12. doi: 10.1093/rheumatology/39.suppl_2.3 11276800

[6] Morrissey AM, O’Neill A, O’Sullivan K, Robinson K. Complementary and alternative medicine use among older adults with musculoskeletal pain: findings from the European Social Survey (2014) special module on the social determinants of health. Br J pain. 2022;16: 109–118. doi: 10.1177/20494637211023293 35111319 PMC8801684

[7] Zochling J, March LM, Lapsley H, Cross M, Tribe K, Brooks P. Use of complementary medicines for osteoarthritis—A prospective study. Ann Rheum Dis. 2004. doi: 10.1136/ard.2003.010637 15082486 PMC1754991

[8] Posadzki P, Ernst E. Osteopathy for musculoskeletal pain patients: a systematic review of randomized controlled trials. Clin Rheumatol 2010 302. 2010;30: 285–291. doi: 10.1007/S10067-010-1600-6 21053038

[9] Lorenc A, Feder G, Macpherson H, Little P, Mercer SW, Sharp D. Scoping review of systematic reviews of complementary medicine for musculoskeletal and mental health conditions. BMJ Open. 2018;8: e020222. doi: 10.1136/BMJOPEN-2017-020222 30327397 PMC6196876

[10] Kocyigit BF, Sagtaganov Z, Yessirkepov M, Akyol A. Assessment of complementary and alternative medicine methods in the management of ankylosing spondylitis, rheumatoid arthritis, and fibromyalgia syndrome. Rheumatol Int. 2022 [cited 28 Jan 2023]. doi: 10.1007/S00296-022-05267-1 36583800 PMC9801164

[11] Barnes PM, Powell-Griner E, McFann K, Nahin RL. Complementary and alternative medicine use among adults: United States, 2002. Semin Integr Med. 2004;2: 54–71. doi: 10.1016/j.sigm.2004.07.003 15188733

[12] Jafari A, Zanganeh M, Kazemi Z, Lael-Monfared E, Tehrani H. Iranian healthcare professionals’ knowledge, attitudes, and use of complementary and alternative medicine: a cross sectional study. BMC Complement Med Ther. 2021;21: 1–11. 34592983 10.1186/s12906-021-03421-z PMC8485522

[13] Nejatian M, Alami A, Tehrani H, Lael–Monfared E, Jafari A. Perceptions and personal use of Complementary and Alternative Medicine (CAM) by Iranian health care providers. Complement Ther Clin Pract. 2018;32: 145–150. 30057042 10.1016/j.ctcp.2018.06.002

[14] JUH Annual Reports 2020. 2020. Available: http://hospital.ju.edu.jo/AnnualReports/Forms/Tagreer Hospetal 2020.pdf

[15] Kotrlik J, Higgins C. Organizational research: Determining appropriate sample size in survey research appropriate sample size in survey research. Inf Technol Learn Perform J. 2001;19: 43.

[16] Afifi FU, Wazaify M, Jabr M, Treish E. The use of herbal preparations as complementary and alternative medicine (CAM) in a sample of patients with cancer in Jordan. Complement Ther Clin Pract. 2010;16: 208–212. doi: 10.1016/j.ctcp.2010.05.001 20920804

[17] Wazaify M, Afifi FU, El-Khateeb M, Ajlouni K. Complementary and alternative medicine use among Jordanian patients with diabetes. Complement Ther Clin Pract. 2011;17: 71–75. doi: 10.1016/j.ctcp.2011.02.002 21457894

[18] Wazaify M, Alawwa I, Yasein N, Al-saleh A, Fatma UA. Complementary Therapies in Clinical Practice Complementary and alternative medicine (CAM) use among Jordanian patients with chronic diseases. 2013;19. doi: 10.1016/j.ctcp.2013.03.001 23890462

[19] Barnes PM, Bloom B, Nahin RL. Complementary and Alternative Medicine Use Among Adults and Children: United States, 2007. 2007. 19361005

[20] Disability-adjusted life years (DALYs). [cited 8 Nov 2022]. Available: https://www.who.int/data/gho/indicator-metadata-registry/imr-details/158

[21] Wazaify M, Alawwa I, Yasein N, Al-Saleh A, Afifi FU. Complementary and alternative medicine (CAM) use among Jordanian patients with chronic diseases. Complement Ther Clin Pract. 2013;19: 153–157. doi: 10.1016/j.ctcp.2013.03.001 23890462

[22] Klingberg E, Wallerstedt SM, Torstenson T, Håwi G, Forsblad-d’Elia H. The use of complementary and alternative medicine in outpatients with inflammatory rheumatic diseases in Sweden. Scand J Rheumatol. 2009;38: 472–480. 19922024 10.3109/03009740902994280

[23] Clarke TC, Nahin RL. Use of Complementary Health Approaches for Musculoskeletal Pain Disorders Among Adults: United States, 2012. Natl Heal Stat Reports Number. 2012;98. Available: https://nccih.nih.gov/health/27736632

[24] Fjær EL, Landet ER, McNamara CL, Eikemo TA. The use of complementary and alternative medicine (CAM) in Europe. BMC Complement Med Ther. 2020;20: 1–9. 32252735 10.1186/s12906-020-02903-w PMC7137515

[25] Mbizo J, Okafor A, Sutton MA, Burkhart EN, Stone LM. Complementary and Alternative Medicine Use by Normal Weight, Overweight, and Obese Patients with Arthritis or Other Musculoskeletal Diseases. J Altern Complement Med. 2016;22: 227–236. doi: 10.1089/ACM.2014.0390 26938367

[26] Youn B-Y, Moon S, Mok K, Cheon C, Ko Y, Park S, et al. Use of traditional, complementary and alternative medicine in nine countries: A cross-sectional multinational survey. Complement Ther Med. 2022;71: 102889. doi: 10.1016/j.ctim.2022.102889 36162719

[27] Lapane KL, Sands MR, Yang S, McAlindon TE, Eaton CB. Use of complementary and alternative medicine among patients with radiographic-confirmed knee osteoarthritis. Osteoarthr Cartil. 2012;20: 22–28. doi: 10.1016/j.joca.2011.10.005 22033041 PMC3254852

[28] Cabrerizo S, De La Cruz JP, López-Villodres JA, Muñoz-Marín J, Guerrero A, Reyes JJ, et al. Role of the inhibition of oxidative stress and inflammatory mediators in the neuroprotective effects of hydroxytyrosol in rat brain slices subjected to hypoxia reoxygenation. J Nutr Biochem. 2013;24: 2152–2157. doi: 10.1016/j.jnutbio.2013.08.007 24231104

[29] Chin KY, Pang KL. Therapeutic Effects of Olive and Its Derivatives on Osteoarthritis: From Bench to Bedside. Nutr 2017, Vol 9, Page 1060. 2017;9: 1060. doi: 10.3390/nu9101060 28954409 PMC5691677

[30] Ometto F, Ortolan A, Farber D, Lorenzin M, Dellamaria G, Cozzi G, et al. Mediterranean diet in axial spondyloarthritis: an observational study in an Italian monocentric cohort. Arthritis Res Ther. 2021;23: 1–13. 34416917 10.1186/s13075-021-02600-0 PMC8377333

[31] AL-Snafi AE. Chemical constituents and pharmacological effects of lepidium sativum. Int J Curr Pharm Res. 2019;11: 1–10. 10.22159/ijcpr.2019v11i6.3633

[32] AlBedah AM, El-Olemy AT, Khalil MKM. Knowledge and attitude of health professionals in the Riyadh region, Saudi Arabia, toward complementary and alternative medicine. J Family Community Med. 2012;19: 93. doi: 10.4103/2230-8229.98290 22870412 PMC3410186

[33] Al-Bedah AMN, Elsubai IS, Qureshi NA, Aboushanab TS, Ali GIM, El-Olemy AT, et al. The medical perspective of cupping therapy: Effects and mechanisms of action. J Tradit Complement Med. 2019;9: 90. doi: 10.1016/j.jtcme.2018.03.003 30963043 PMC6435947

[34] Kolasinski SL, Neogi T, Hochberg MC, Oatis C, Guyatt G, Block J, et al. 2019 American College of Rheumatology/Arthritis Foundation Guideline for the Management of Osteoarthritis of the Hand, Hip, and Knee. Arthritis Care Res (Hoboken). 2020;72: 149–162. doi: 10.1002/acr.24131 31908149 PMC11488261

[35] Airaksinen O, Brox JI, Cedraschi C, Hildebrandt J, Klaber-Moffett J, Kovacs F, et al. Chapter 4. European guidelines for the management of chronic nonspecific low back pain. Eur Spine J. 2006;15 Suppl 2: S192–300. doi: 10.1007/s00586-006-1072-1 16550448 PMC3454542

[36] Oliveira CB, Maher CG, Pinto RZ, Traeger AC, Lin CWC, Chenot JF, et al. Clinical practice guidelines for the management of non-specific low back pain in primary care: an updated overview. Eur Spine J. 2018;27: 2791–2803. doi: 10.1007/s00586-018-5673-2 29971708

[37] A Q TJW, RM M, MA F, TD D, MJ B, et al. Noninvasive Treatments for Acute, Subacute, and Chronic Low Back Pain: A Clinical Practice Guideline From the American College of Physicians. Ann Intern Med. 2017;166: 514–530. doi: 10.7326/M16-2367 28192789

[38] Bardaweel SK, Shehadeh M, Suaifan GARY, Kilani MVZ. Complementary and alternative medicine utilization by a sample of infertile couples in Jordan for infertility treatment: Clinics-based survey. BMC Complement Altern Med. 2013;13: 1–7. doi: 10.1186/1472-6882-13-35/FIGURES/5 23414246 PMC3599001

[39] Bank W. Jordan Country Reclassification—Questions and answers. 2017.

[40] Ababsa M. The socio-economic composition of the population. Beirut, Lebanon Press l’Ifpo. 2013.

[41] Herman PM, Poindexter BL, Witt CM, Eisenberg DM. Are complementary therapies and integrative care cost-effective? A systematic review of economic evaluations. BMJ Open. 2012;2. doi: 10.1136/bmjopen-2012-001046 22945962 PMC3437424

[42] Gohil NJ, Patel VJ. Use of complementary and alternative medicines by patients with orthopaedic disorders in western part of India: a cross sectional study. Int J Basic Clin Pharmacol. 2017;5: 1722–1726. doi: 10.18203/2319-2003.IJBCP20163206

[43] Shehadeh MB, Suaifan G, Abu-Odeh A, Darwish R. Complementary and alternative medicine use for weight management among females in jordan: A community-based survey. East Mediterr Heal J. 2020;26: 443–452. doi: 10.26719/EMHJ.19.098 32338363

[44] Gerber LM, Mamtani R, Chilu YL, Bener A, Murphy M, Cheema S, et al. Use of complementary and alternative medicine among midlife Arab women living in Qatar. East Mediterr Health J. 2014;20: 554. doi: 10.26719/2014.20.9.554 25343468 PMC4364539

[45] Phang JK, Kwan YH, Goh H, Tan VIC, Thumboo J, Østbye T, et al. Complementary and alternative medicine for rheumatic diseases: A systematic review of randomized controlled trials. Complement Ther Med. 2018;37: 143–157. doi: 10.1016/j.ctim.2018.03.003 29609927

[46] Danve A, Deodhar AA. Complementary medicine for axial spondyloarthritis: is there any scientific evidence? Curr Opin Rheumatol. 2018;30: 310–318. doi: 10.1097/BOR.0000000000000513 29634580

[47] Sprague S, Lutz K, Bryant D, Farrokhyar F, Zlowodzki M, Bhandari M. Complementary and alternative medicine use in patients with fractures. Clin Orthop Relat Res. 2007;463: 173–178. 17960679

[48] Cinar FI, Sinan O, Yilmaz S, Bagcivan G, Aydogan I, Yalcin AG, et al. Use of complementary and alternative medicine in patients with ankylosing spondylitis. Eur J Rheumatol. 2021;8: 20–26. doi: 10.5152/eurjrheum.2020.20111 33196421 PMC7861640

[49] Grainger R, Walker J. Rheumatologists’ opinions towards complementary and alternative medicine: a systematic review. Clin Rheumatol. 2014;33: 3–9. doi: 10.1007/s10067-013-2379-z 23990027

