# Supplementary material for: Rates, patterns, and predictors of complementary medicine use among patients with musculoskeletal diseases
Source: PLoS One. 2023 Jun 23;18(6):e0287337. doi: 10.1371/journal.pone.0287337 (PMC10289458; doi:10.1371/journal.pone.0287337)
Supplement: S1 File — (PDF) [file pone.0287337.s001.pdf]

Use of Complementary and Alternative medicines in Arthritis patients in Jordan.

Study ID: CAM

Patient's name	MRN:	MD: FA/ MH/JA

Section A:
Demographic Details of the patient:

**Gender**	❑ Male ❑ Female
**Age group (years)**	❑ < 16 ❑ 16-25 ❑ 26- 40 ❑ 41-50 ❑ 51-60 ❑ > 60
**Marital status**	❑ single ❑ married ❑ Divorced ❑ Widow
**Next of kin (NOK) :**	❑N/A ❑Yes Relation to patient: -----
**Occupation**	……………………………………
**Place of residence**	❑ Amman ❑ Other Specify area of residence……………………
**Area**	❑Urban ❑ Rural
**Educational level**	❑ Illiterate ❑ Elementary school ❑ High school ❑ Diploma ❑ Bachelor degree ❑ Graduate studies (MSc. Ph.D) ❑ Other………………
**Monthly income**	❑ <400 JD ❑ 400-1000JD ❑ >1000JD

Section B:<br>History of present illness:	
**Musculoskeletal diagnosis:**	………………..
**Time of diagnosis:**	…………………
**Other medical problems (e.g. hypertension, diabetes, heart disease, thyroid, asthma, cancer, others)**	…………………
**Patient Medications List**:<br>(Obtained either from interview or from patient file)	………………………………
**Reason for this visit:**<br>……………………..	❑First visit<br>❑Follow up visit

Use of Complementary and Alternative medicines in Arthritis patients in Jordan.

Study ID: CAM

Have you ever used any of the following?

(البصل ، الشيح، البردقوش، الثوم ، الصبر الحقيقي، غالنجال، الشاي الأخضر، حبة البركة ، القرفة، بذور الكتان، زيت الزيتون، الحلبة، الكركم، الزنجبيل، العسل، حب الرشاد، الكولاجين، زيت السمك ، ماء زمزم، فيتامينات ، *Chondroitin /glucosamine*)

CAM TYPE	**Do you use any of These CAM?**			**In which form do you use It?**						**Where did you Get it?**		**DOSE**	**How often do you use such remedies?**				**How do you use this CAM**	
	Ever use	Current use	Never Use	Crude extract	Dosage form	Tea	Spices	Injections	others	Jordan	Abroad		Daily	weekly	Only once	occasionally	Along with conventional treatment	instead of it

Use of Complementary and Alternative medicines in Arthritis patients in Jordan.

Study ID: CAM

CAM TYPE	How long have you been using such remedies? (months)	Who recommended the use of such remedies?						Why do you take this remedy?					Have you got the sought effect?		Are you aware of any side effects of the CAM used?	
		friend	family member	doctor	pharmacist	herbalist	Media	Improve general health	Cure of disease	Slow down progression of disease	Relieve symptoms of disease	Reduce side effects of medication	yes	no	yes	No

CAM TYPE	Do you think this remedy is beneficial for you?					Did you discuss using such remedies with the doctor? (yes)			Did you discuss using such remedies with the doctor? (no)					How much does it cost you to get such remedies (per month if frequent use or per single treatment if occasional use)?				
	not at all satisfied	slightly satisfied	moderately satisfied	Very satisfied	completely satisfied	Ok to use	Not to use	Up to you to use it or not	Afraid he/she will stop treating me	Afraid he/she will get mad on me	The doctor didn't ask me	No reason to mention	others	<10	25-50	50-100	100-300	>300

Use of Complementary and Alternative medicines in Arthritis patients in Jordan.

Study ID: CAM

Have you ever used any of the following?

Heat and massage , Water therapy , Acupuncture, Cupping, Henna, Bees stings , Religious and spiritual healing (Prayers, supplication, reading Quran) , Bracelets(Wool , magnets, copper), IA Platelet rich plasma , IA Stem cells, Gluten free diet, Elimination diet, Food avoided because of MSK disease (name it)

Others (name it)

CAM TYPE	**Do you use any of These CAM?**			**In which form do you use It?**						**Where did you Get it?**		**DOSE**	**How often do you use such remedies?**				**How do you use this CAM**	
	Ever use	Current use	Never Use	Crude extract	Dosage form	Tea	Spices	Injections	others	Jordan	Abroad		Daily	weekly	Only once	occasionally	Along with conventional treatment	instead of it

Use of Complementary and Alternative medicines in Arthritis patients in Jordan.

Study ID: CAM

CAM TYPE	**How long have you been using such remedies? (months)**	**Who recommended the use of such remedies?**						**Why do you take this remedy?**					**Have you got the sought effect?**		**Are you aware of any side effects of the CAM used?**	
		friend	family member	doctor	pharmacist	herbalist	Media	Improve general health	Cure of disease	Slow down progression of disease	Relieve symptoms of disease	Reduce side effects of medication	yes	no	yes	No

CAM TYPE	**Do you think this remedy is beneficial for you?**					**Did you discuss using such remedies with the doctor? (yes)**			**Did you discuss using such remedies with the doctor? (no)**					**How much does it cost you to get such remedies (per month if frequent use or per single treatment if occasional use)?**				
	not at all satisfied	slightly satisfied	moderately satisfied	Very satisfied	completely satisfied	Ok to use	Not to use	Up to you to use it or not	Afraid he/she will stop treating me	Afraid he/she will get mad on me	The doctor didn't ask me	No reason to mention	others	<10	25-50	50-100	100-300	>300
